# Effectiveness of Online Mindfulness-Based Interventions on Psychological Distress and the Mediating Role of Emotion Regulation

**DOI:** 10.3389/fpsyg.2018.02090

**Published:** 2018-10-31

**Authors:** Ying Ma, Zhaozhuo She, Angela Fung-Ying Siu, Xianglong Zeng, Xinghua Liu

**Affiliations:** ^1^School of Education, Shaanxi Normal University, Xi’an, China; ^2^Beijing Key Laboratory of Learning and Cognition, Capital Normal University, Beijing, China; ^3^Faculty of Education, The Chinese University of Hong Kong, Shatin, Hong Kong; ^4^Faculty of Psychology, Beijing Normal University, Beijing, China; ^5^School of Psychological and Cognitive Sciences, Peking University, Beijing, China

**Keywords:** online mindfulness-based intervention, psychological distress, emotion regulation, randomized control trial, mediating effect

## Abstract

Online mindfulness-based intervention as a feasible and acceptable approach has received mounting attention in recent years, yet more evidence is needed to demonstrate its effectiveness. The primary objective of this study was to examine the effects of online mindfulness-based programs on psychological distress (depression and anxiety). The randomized controlled intervention design consisted of four conditions: group mindfulness-based intervention (GMBI), self-direct mindfulness-based intervention (SDMBI), discussion group (DG) and blank control group (BCG). The program lasted 8 weeks and a total of 76 participants completed the pre- and post-test. Results showed that participants in GMBI and SDMBI had significant pre- and post-test differences on mindfulness, emotion regulation difficulties, and psychological distress, with medium to large effect sizes. In addition, ANCOVA results indicated significant effects of group membership on post-test scores of mindfulness, depression and anxiety when controlling the pretest scores, with medium to large effect sizes. The GMBI appeared to exert the greatest effects on outcome variables in comparison with other groups. In addition, changes in emotion regulation difficulties across groups could mediate the relationship between changes in mindfulness dimensions (Observing and Describing) and changes in psychological distress across groups. These results provided encouraging evidence for the effectiveness of online mindfulness-based interventions in reducing psychological distress, and the possible mediating role of emotion regulation, while also underlining the importance of group discussion in online mindfulness-based interventions.

## Introduction

Mindfulness was defined as “the awareness that emerges through paying attention, on purpose, in the present moment, and non-judgmental to the unfolding of experience moment by moment” (Kabat-Zinn, 2003, p. 145). Since late 1970s, mindfulness-based interventions (MBIs) such as Mindfulness-Based Stress Reduction (MBSR) program (Kabat-Zinn, 1990) and Mindfulness-Based Cognitive Therapy (MBCT) (Segal et al., 2002) have been widely used to enhance psychological wellbeing in both clinical and non-clinical samples (e.g., Hofmann et al., 2010; Spijkerman et al., 2016). Both MBSR and MBCT are 8-session group-based therapies which incorporate mindfulness practices with other therapy approaches of stress reduction and cognitive reappraisals. Many previous studies have proven the effectiveness of MBIs in helping to improve life satisfaction and positive emotions (Sears and Kraus, 2009; Grossman et al., 2010), and to reduce psychological distress such as depression and anxiety (Goldin and Gross, 2010; Boettcher et al., 2014; Khoury et al., 2015).

In recent years, there is a growing number of online interventions targeting many different symptoms and conditions for various population groups (Andersson and Cuijpers, 2008; Currie et al., 2010; Boettcher et al., 2014). For example, the study of Currie et al. (2010) found that an Internet-based cognitive behavioral therapy-based program could help reduce emotional distress of college students. Research also indicated that online MBIs could provide a more accessible and easily disseminated approach to deliver mindfulness-based programs to large groups (Kvillemo et al., 2016; Wahbeh and Oken, 2016). The first review and meta-analysis study on the effectiveness of online MBIs in improving mental health found that online MBIs had small but significant beneficial impact on mindfulness and psychological distress including stress, anxiety and depression (Spijkerman et al., 2016). To contribute to a better understanding of the effectiveness of online MBIs, more random control design studies are still needed.

The existing studies on online mindfulness programs are mostly group-based MBIs, and a few studies of self-direct MBIs also showed early promise (Cavanagh et al., 2013, 2014). Compared with self-direct MBIs, group-based MBIs not only include content and practice focusing on the cultivation of mindfulness, but also provide an environment where participants could share an enhanced sense of community and feel supported by each other (Lewis et al., 2012), since social support can play a critical role in traditional group-based MBIs (Malpass et al., 2012). The study of Schellekens et al. (2017) confirmed that increased social support played an important mediating role in the effects of mindfulness intervention on mood disturbance and stress symptoms. Thus, the combination of mindfulness practice with group support may have greater efficacy in helping to reduce psychological distress than self-direct MBIs. Given the rapid expansion of online mental program and easy accessibility, online self-direct MBIs in recent years begin attracting more attention and showing early promising effectiveness (Cavanagh et al., 2014). Significant benefits of self-direct MBIs for mindfulness skills and for symptoms of anxiety and depression were found in some previous studies (Lewis et al., 2012; Cavanagh et al., 2013). Some reviews and meta-analyses have indicated that self-direct intervention may be beneficial to people experiencing common problems such as anxiety and depression (Coull and Morris, 2011; Lewis et al., 2012). However, the comparison of self-direct interventions with therapist-administered interventions showed the latter with a larger effect size (Lewis et al., 2012). Although self-direct MBIs could help reduce psychological distress and allow time flexibility for the arrangement of weekly sessions, participants still reported the experience of lack of support (Kvillemo et al., 2016). The removal of the group context may be a disadvantage to self-direct MBIs (Cavanagh et al., 2014). As self-guided MBI might provide greater reach and cost effectiveness but also with some limits, thus, more evidence is needed to extend this small, but promising research field.

To compare the effectiveness of MBIs with other therapies, random control design is necessary to help identify the special contribution of MBI. Some previous studies have compared the effectiveness of MBIs with other active control groups. For example, Carlson et al. (2015) conducted a research to examine the effectiveness of mindfulness-based cancer recovery and supportive-expressive group therapy to help cancer survivors relieve distress. Their results suggested that both mindfulness group and supportive-expressive group could help participants reduce distress. Schellekens et al. (2017) also found that MBSR participants showed significant improvements on mood disturbance, stress symptoms and social support compared with the supportive-expressive group. To our knowledge, the online mindfulness programs are still in an early pilot phase and require more random control design studies. Therefore, it’s important to examine the different levels of effectiveness of online group mindfulness-based intervention (GMBI) and self-direct mindfulness-based intervention (SDMBI), when compared with other active control groups such as discussion group (DG).

Previous studies have provided initial evidence of emotional regulation ability as an underlying mechanism of MBIs (Goldin and Gross, 2010; Gratz and Tull, 2010). Whether emotion regulation could serve as a special mechanism underlying MBIs compared with other conditions still calls for more investigation. Emotion regulation refers to the ability to manage affective states effectively and is identified as a critical cause of many psychological problems (Gross, 1998). Poor emotional awareness, inappropriate expression of negative emotions and maladaptive coping strategies are predictive of high depressive and anxious symptoms (D’Avanzato et al., 2013). Previous studies also demonstrated that emotion regulation difficulties had a significant relationship with negative affects including depression and anxiety (Vujanovic et al., 2008; Gratz and Tull, 2010). Mindfulness was confirmed to be positively related to adaptive emotion regulation processes in both clinical and non-clinical populations (Roemer et al., 2009; Gratz and Tull, 2010; Pepping et al., 2016). The research of Goldin and Gross (2010) found that participants in the mindfulness practice such as breath focused attention task showed diminished negative emotion experience, reduced amygdala activity, and increased activity in brain regions related to attentional deployment. Mindfulness training could strengthen individuals’ ability to monitor their internal reactions in emotion-eliciting situations and thereby realize when they are in the grip of emotions and need to take time to calm down before responding. The role of non-judgment in mindfulness could facilitate the capacity to view one’s emotional experience from a more objective perspective. In addition, individuals who undertake mindfulness training are taught how to cultivate an attitude of kindness and compassion toward themselves, especially during moments of difficulties (Gratz and Tull, 2010). All these core components of mindfulness could effectively help disrupt the maladaptive and automatic reactions on one’s emotions (Gratz and Tull, 2010). Vujanovic et al. (2010) found that greater levels of the mindfulness skills such as observing, describing, acting with awareness, and accepting without judgment were associated with fewer emotion regulation difficulties (e.g., emotional avoidance and lack of emotional awareness). Therefore, the role of emotion regulation may serve as a mediator to explain the effectiveness of online MBIs on psychological distress compared with control groups.

In conclusion, the main objectives of the present study were to investigate the effectiveness of online MBIs on psychological distress and the possible mediating role of emotion regulation. We would like to compare the effectiveness of online GMBI and SDMBI with DG and blank control group (BCG) to evaluate the effectiveness of online MBIs and further identify the active components of this intervention approach. We proposed three hypotheses: (1) Participants in GMBI, SDMBI would have significant improvement in mindfulness, and significant reductions of emotion regulation difficulties and psychological distress compared with BCG. And GMBI would have stronger effects on outcome variables than SDMBI. (2) Participants in DG would have significant decreases in psychological distress, but no significant differences in mindfulness and emotion regulation difficulties compared with BCG. (3) Changes in emotion regulation difficulties could mediate the effects of online MBIs on psychological distress.

## Materials and Methods

### Participants and Procedures

**FIGURE 1:**
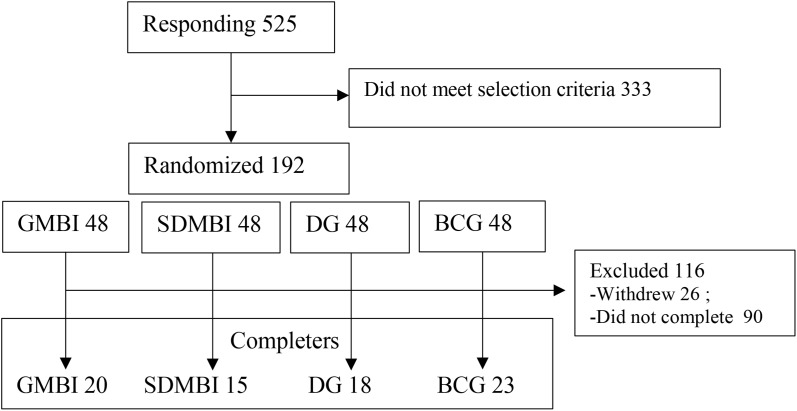
Participants recruitment process.

A total of 525 potential volunteers responded to the web-based advertisements. With the original selective criteria, these individuals needed to join the study on a voluntary basis with the aim of relieving stress, have access to computers and Internet and could understand instructions in Chinese. Additionally, they should not have any prior mindfulness or meditation experience. They had self-claimed to be mentally healthy without identified mental illness. At last, 192 participants enrolled in the program, completed the informed consent and pretest. All participants were randomly assigned to different online groups including GMBI, SDMBI, DG, and BCG. Each group was originally assigned 48 participants. Participants in each group were asked to complete a questionnaire before and after the program. The participant’s recruitment process was shown in Figure 1.

In group of GMBI, nine participants withdrew after the first session, and 19 participants absent more than four sessions were excluded. At last, there were 20 participants completing the pre- and post-tests. In SDMBI, 32 participants who did not submit weekly report more than four times were excluded. One participant who did not complete the post-test was also excluded. In DG, 17 participants withdrew after the first session, and 13 participants who were absent more than four times were excluded. In BC, 25 participants who did not complete the post-test were excluded.

In all, a total 76 participants completed both pre- and post- tests after the whole program, with 44 females and 32 males. The age range was from 18 to 47 (*M* = 27.84, *SD* = 7.94). Table 1 displays sociodemographic characteristics of different intervention conditions.

**Table 1:** Content of online mindfulness-based intervention.

Session	Content	Practice
1	***Awareness and Automatic Pilot:*** Recognize the tendency of automatic pilot, become aware of each moment	Body and breathing sensation
2	***Living in Our Heads:*** further focus on body sensation, tend to control reactions to daily events, learn about emotion	Further body scan
3	***Gathering the Scattered Mind:*** Recognize how the mind can often be busy and scattered, taking awareness to breath and movement	Breathing space; mindful stretching; mindful walking
4	***Recognizing the Territory of Aversion:*** Take a different and wider perspective to experience, know the territory of depression	Sounds and thoughts meditation; difficulties exploration
5	***Allowing/Letting Be:*** cultivate attitudes of non-judgment and acceptance	Body scan and breathing space
6	***Thoughts Are Not Facts:*** Recognize the thoughts related to our experience, and work with thoughts with curiosity and kindness	Kindness mediation
7	***How Can I Best Take Care of Myself:*** Learn how to deal with negative emotion threatens, make plans to respond to the signs	Breathing space; mindfulness bells
8	***Maintaining and Extending New Learning:*** Recognize mindfulness could help balance the life, take care of oneself	Keep mindfulness in daily life


### Intervention Process

The intervention implemented in the present study was a revision of MBCT. The instructor is a master majoring in Counseling Psychology and with 3 years’ solid experience of mindfulness intervention. In the GMBI group, the intervention consisted of eight 2-h weekly sessions. In each session, there was 40-min mindfulness-based practice. In the remaining time, the group members discussed their experience and homework in the previous week. The audio practices were distributed to participants after each weekly session. The intervention content mainly contained mindfulness practices and some cognitive therapy elements. Formal mindfulness practices employed included body scan, breathing space, mindful sitting, mindful stretch, etc. Cognitive therapy elements included, for example, how to recognize the thoughts related to our experience and take a different and wider perspective to experience. Compared with the traditional MBCT, this online GMBI did not include the 1-day retreat due to the limitation of the online environment. In addition to the recording of happiness and unhappiness as in the second and third weeks of the traditional MBCT, this GMBI also included an assignment of recording events of stress and communications in the fourth and fifth weeks. The content of online MBI in present study was shown in Table 1.

Participants in the SDMBI group only received the materials and practice guidance without group discussion sessions. The materials were the same as that of the GMBI group including mindfulness related reading material and practice audio. These self-directed materials were distributed to participants every week. Participants were asked to report their practice time and experience on a weekly basis.

Participants in DG engaged in a closed and supervised online discussion forum. The topics they discussed were associated with emotion events. For instance, topics included positive and negative events, stress, and interpersonal communications, as well as how the participants perceived their psychological distress such as stress, anxiety, and depression symptoms, and how they dealt with their emotional problems. These online dialogs were supervised by an instructor who did not play an active role in the discussions.

Participants in BCG received no intervention. They were informed that they could join the online MBI in another cohort.

### Measures

Five Facet Mindfulness Questionnaire (FFMQ) (Baer et al., 2008) is a 39-item questionnaire which includes five facets of mindfulness: Observing, Describing, Acting with awareness, Non-judging of inner experience, and Non-reactivity to inner experience. Items were rated on a 5-point metric of frequency (1 = almost never and 5 = almost always). A higher total score means a higher level of mindfulness. The Chinese version of FFMQ developed by Deng et al. (2011) was used in this study. Cronbach’s alphas for our sample were 0.92 (pre) and 0.93 (post).

Difficulties in Emotion Regulation Scale (DERS) (Gratz and Roemer, 2004) is a 36-item Likert-type scale. Participants indicated how often the items applied to themselves, with responses ranging from 1 (almost never) to 5 (almost always). There are six factor structure of the DERS including lack of emotional awareness (AWARENESS), lack of emotional clarity (CLARITY), difficulty in engaging in goal-direct behavior under negative emotions (GOALS), loss of control under negative emotions (IMPULSE), limited strategies for emotion regulation (STRATEGIES), and non-acceptance of emotional responses (NON-ACCEPTANCE) (Gratz and Roemer, 2004). The total score of DERS was generally suggested to be used in previous studies to present the total dysfunction in emotion regulation. The Chinese version of the DERS had demonstrated good reliability and validity (Wang et al., 2007). Cronbach’s alphas for our sample were 0.96 (pre) and 0.95 (post).

The Self-Rating Anxiety Scale (SAS) (Zung, 1971) was used to assess anxiety symptoms. Each instrument includes 20 items in a four-point scale ranging from 1 (never) to 4 (always), with total scores ranging from 20 to 80. A higher total score denotes a higher level of anxiety. The Chinese version of SAS has been used in many previous studies (e.g., Liu et al., 1999). Cronbach’s alphas for internal consistency reliabilities for our sample were 0.87 (pre) and 0.90 (post).

The Self-Rating Depression Scale (SDS) (Zung, 1965) was used to assess depression symptoms. This scale includes 20 items, which is rated from 1 (never) to 4 (always), with total scores ranging from 20 to 80. A higher total score denotes a higher level of depression. The Chinese version of SDS has been used in many previous studies (e.g., Liu et al., 1999; Yu et al., 2015). Cronbach’s alphas for internal consistency reliabilities for our sample were 0.88 (pre) and 0.89 (post).

### Data Analyses

Preliminary analyses were conducted to determine whether the four groups differ in the pre-program period.

The main purpose of this study was to explore if the online MBIs and control groups would evolve differently throughout the program, by comparing the results of the pretest to posttest. To tackle this question, we first used paired-samples *t-*test to compare the differences between the pretest and posttest outcome variables in each group. The effect sizes were calculated through Cohen’s *d* which was recommended with values of 0.20, 0.40, and 0.60, indicating effect sizes of small, medium, and large (Cohen, 1988). Then ANCOVAs, which was a general method best suited to examine between-groups differences of pretest to posttest in a randomized control design (Huck and McLean, 1975; Perez-Blasco et al., 2013), were employed to answer whether the four groups’ posttest means would differ after controlling the pretest scores. The $\eta_{p}^{2}$ was included as an indicator of effect size with approximate values of 0.01, 0.06, and 0.14, indicating effect sizes of small, medium, and large (Cohen, 1988; Perez-Blasco et al., 2013).

Finally, to calculate the mediating role of emotion regulation of the intervention effect, the indirect effects were estimated using SPSS process (Preacher and Hayes, 2008) and then bootstrapping procedure was also used to examine the significance of indirect effect. The bias corrected and accelerated 95% confidence intervals were then examined, and if these intervals did not contain zero, the point estimate of the indirect effect would be considered significant. For all the analyses, the level of statistical significance was set to 0.05.

## Results

The results of chi-square test and one-way ANOVAs indicated that participants in the four intervention conditions were not significantly different prior to the intervention in gender or age (Table 2). Table 3 shows descriptive statistics of mindfulness, difficulties of emotion regulation, depression and anxiety in pre- and post-tests. No significant differences were found in any of the outcome variables prior to the intervention, including FFMQ [*F*(3,72) = 0.24; *p* = 0.87], DERS [*F*(3,72) = 0.19; *p* = 0.91], SDS [*F*(3,72) = 0.95; *p* = 0.42], SAS [*F*(3,72) = 0.42; *p* = 0.74].

**Table 2:** Demographic characteristics of participants.

	GMBI (*n* = 20)	SDMBI (*n* = 15)	DG (*n* = 18)	BCG (*n* = 23)		*p*
Male	7	8	8	9	χ^2^= 1.31	0.73
Female	13	7	10	14		
Age *M (SD)*	29.15 (8.22)	29.47 (9.17)	26.39 (6.85)	26.78 (7.78)	*F* = 0.72	0.54


*GMBI, group mindfulness-based intervention; SDMBI, self-direct mindfulness-based intervention; DG, discussion group; BCG, blank control group.*

**Table 3:** Descriptive statistics, *t*-test and ANCOVA results for the studied variables.

	GMBI			SDMBI			DG			BCG			ANCOVA
	Pretest	Posttest	*t*	Pretest	Posttest	*t*	Pretest	Posttest	*t*	Pretest	Posttest	*t*	*F*(3,71)
	*M*	*M*	*(d)*	*M*	*M*	*(d)*	*M*	*M*	*(d)*	*M*	*M*	*(d)*	($\eta_{p}^{2}$)
	*(SD)*	*(SD)*		*(SD)*	*(SD)*		*(SD)*	*(SD)*		*(SD)*	*(SD)*		
FFMQ	112.93	130.14	-3.57^∗∗^	108.15	121.72	-2.62^∗^	113.12	123.22	-3.57^∗∗^	113.84	117.02	-1.29	3.22^∗^
	(24.43)	(26.00)	(0.68)	(13.77)	(16.03)	(0.91)	(20.53)	(14.77)	(0.56)	(20.32)	(16.34)	(0.17)	(0.12)
DERS	103.60	90.51	2.69^∗^	106.67	93.88	3.40^∗∗^	99.78	94.47	1.02	103.10	100.47	0.62	1.87
	(27.50)	(25.35)	(0.49)	(20.15)	(15.83)	(0.71)	(32.39)	(17.35)	(0.21)	(23.04)	(17.82)	(0.13)	(0.07)
SDS	38.98	32.11	3.62^∗^	43.12	39.39	2.04^∗^	42.80	38.25	2.19^∗^	42.84	40.02	1.65	2.93^∗^
	(10.04)	(8.24)	(0.75)	(6.71)	(8.66)	(0.48)	(10.14)	(7.73)	(0.50)	(7.45)	(7.89)	(0.37)	(0.11)
SAS	36.17	31.71	1.88	39.22	38.61	0.41	38.65	34.46	2.60^∗^	38.83	39.50	-0.57	4.83^∗∗^
	(10.91)	(9.34)	(0.44)	(5.69)	(7.23)	(0.09)	(10.05)	(6.70)	(0.49)	(8.44)	(8.44)	(0.08)	(0.17)


*^∗^p < 0.05, ^∗∗^p < .01. GMBI, group mindfulness-based intervention; SDMBI, self-direct mindfulness-based intervention; DG, discussion group; BCG, blank control group; FFMQ, Five Facet Mindfulness Questionnaire; DERS, Difficulties in Emotion Regulation Scale; SDS, Self-Rating Depression Scale; SAS, Self-Rating Anxiety Scale.*

### Changes in the Outcome Variables From Pre- to Post-test

Comparisons of the pre- and post-test scores of outcome variables across different groups are presented in Table 3. In the GMBI group, the score of FFMQ surged considerably from pre- to post-test, while scores of DERS and SDS dropped remarkably, with medium to large effect sizes in Cohen’s *d*. In the SDMBI group, there were significant pre- and post-test changes in scores of FFMQ, DERS, and SDS. In DG, there were also significant increase in the score of FFMQ, and significant decrease in SDS and SAS, but no notable changes in DERS. In the BCG group, data analysis revealed no statistically changes in levels of all studied variables.

### Differences in Posttest Scores on Outcome Variables Between Groups

The one-way analysis of covariance (ANCOVA) was used to identify if there were between-group differences on posttest levels of FFMQ, DERS, SDS, and SAS after controlling the pretest levels of these variables. Homogeneity of regression assumption was not violated for these variables. There were no interactions between any of the covariates (Pretest measures of FFMQ, DERS, SDS, and SAS) and the group membership [pretest FFMQ × group membership, *F*(3,68) = 1.14, *p*(3,68) = 0.34; pretest DERS × group membership, *F*(3,68) = 0.58, *p* = 0.63; pretest SDS × group membership, *F*(3,68) = 0.43, *p* = 0.73; pretest SAS × group membership, *F*(3,68) = 0.79, *p* = 0.50].

After controlling the pretest levels of FFMQ, DERS, SDS, SAS scores separately, there were significant effect of group membership on the posttest levels of FFMQ, SDS, and SAS, but the effect on DERS was not remarkable (Table 3). While the effect sizes (η^2^) of all the outcome variables were from medium to large (0.07 to 0.17), which suggested potential effects of group membership on all the posttest outcome variables.

The *post hoc* tests were run to make pairwise comparisons of adjusted mean scores among all outcome variables (Table 4). The adjusted mean score of posttest FFMQ was significantly higher for participants in GMBI than those in BCG. And the adjusted mean scores of posttest DERS, SDS and SAS were significantly lower for participants in the GMBI compared with those in the BCG. Participants in DG showed significantly lower adjusted mean score of SAS than those in BCG. The adjusted mean scores of other outcome variables in SDMBI and DG didn’t show significant differences when compared with BCG.

**Table 4:** Pairwise comparisons of adjusted mean scores of outcome variables.

Outcome variables		GMBI *MD (SE)*	SDMBI *MD (SE)*	DG *MD (SE)*
FFMQ	SDMBI	5.52 (4.97)	-	-
	DG	7.02 (4.71)	1.51(5.08)	-
	BCG	13.66* (4.43)	8.15 (4.84)	6.64 (4.56)
DERS	SDMBI	-1.88 (5.51)	-	-
	DG	-5.81 (4.90)	-3.93 (5.29)	-
	BCG	-6.03* (4.61)	-8.32 (5.01)	-4.39 (4.75)
SDS	SDMBI	-5.26* (2.40)	-	-
	DG	-4.28 (2.28)	0.98 (2.43)	-
	BCG	-6.03** (2.15)	-0.77 (2.30)	-1.75 (2.18)
SAS	SDMBI	-5.32* (2.13)	-	-
	DG	-1.47 (2.02)	3.85 (2.17)	-
	BCG	-6.42** (1.91)	-1.09 (2.06)	-4.95* (1.95)


*^∗^p < 0.05, ^∗∗^p < 0.01. MD, mean difference; SE, standard error; GMBI = group mindfulness-based intervention; SDMBI, self-direct mindfulness-based intervention; DG, discussion group; BCG, blank control group; FFMQ, Five Facet Mindfulness Questionnaire; DERS, Difficulties in Emotion Regulation Scale; SDS, Self-Rating Depression Scale; SAS, Self-Rating Anxiety Scale.*

### Emotion Regulation as Mediator

As reported in the above analysis of intervention effects, there was no significant group membership effect on DERS; therefore, we did not include group membership in the mediation analysis. Correlations between changes of outcome variables across groups appear in Table 5. The changes in total score and subscales of FFMQ were all negatively related to the change in DERS across groups. And the score change of DERS was positively related to changes in SAS and SDS across groups. The score changes of FFMQ, DERS, SAS, and SDS in mediation analysis had also been examined. Results of mediating effects analysis through SPSS process (Preacher and Hayes, 2008) indicated that there was no significant indirect effect of change in FFMQ on changes in SAS (*B* = -0.114, SE = 0.096; 95% CI = [-0.422, 0.004]) and SDS (*B* = -0.005, SE = 0.015; 95% CI = [-0.038, 0.023]) through the mediating role of change in DERS. Then a similar analysis using the FFMQ subscales instead of the FFMQ total score was conducted. The results found that there were significant indirect effects of changes in the dimension of Describing on changes in SAS (*B* = -0.099, SE = 0.076; 95% CI = [-0.325, -0.002]), and SDS (*B* = -0.118, SE = 0.089; 95% CI = [-0.365, -0.003]). The change in the dimension of Observing in change of SAS (*B* = -0.099, SE = 0.076; 95% CI = [-0.325, -0.002]) also exerted a significant indirect effect.

**Table 5:** Descriptive statistics, correlations between changes in studied variables across groups.

	1	2	3	4	5	6	7	8	9
(1) FFMQ	-								
(2) Observing	0.30**	-							
(3) Describing	0.26*	0.52**	-						
(4) Acting with awareness	0.33**	0.29**	0.37**	-					
(5) Non-judging inner exp.	0.30**	0.14	0.23*	0.46**	-				
(6) Non-reactivity inner exp.	0.20	0.51**	0.32**	0.45**	0.26*	-			
(7) DERS	-0.35**	-0.30**	-0.25*	-0.47**	-0.29*	-0.42**	-		
(8) SAS	-0.59**	-0.23**	-0.09	-0.26**	-0.33**	-0.22	0.27*	-	
(9) SDS	-0.63**	-0.29**	-0.03	-0.27*	-0.38**	-0.19	0.25*	0.74**	-


*^∗^p < 0.05, ^∗∗^p < 0.01. FFMQ, Five Facet Mindfulness Questionnaire; DERS, Difficulties in Emotion Regulation Scale; SDS, Self-Rating Depression Scale; SAS, Self-Rating Anxiety Scale.*

## Discussion

With a random control design, the current study investigated the effectiveness of online MBIs on psychological distress of general population seeking stress reduction. Consistent with the hypotheses, online MBIs showed promising effectiveness on the reduction of anxiety and depression. The results also emphasized the important role of group support in online MBI as participants in online MBI within group situation seemed exerting the most significant effectiveness. Additionally, the emotion regulation difficulties could serve as a possible mediating role to help extend our knowledge of the mechanism underlying the effects of MBIs.

There were significant pretest–posttest differences of the level of mindfulness in the GMBI and SDMBI groups. Statistical examination of the group differences of the posttest outcome variables suggested that there were medium to large effect sizes of group membership effects on the posttest level of mindfulness. GMBI showed significant difference in mindfulness compared with BCG group. These results were in line with the limited previous studies suggesting that online MBIs were effective to cultivate mindfulness (Spijkerman et al., 2016). DG also had significant effect on mindfulness, which was out of our hypotheses. One previous study of Schellekens et al. (2017) also found that both MBCT and supportive-expressive group therapy could help improve participants’ level of mindfulness, and MBCT did not show significant improvement on mindfulness compared with supportive-expressive group therapy. A possible reason is that the group discussion about the participants’ positive and negative emotion states and emotion regulation strategies might indirectly help raise their levels of awareness and clarity of their own emotions (Schellekens et al., 2017). Increased awareness and clarity of emotions through group discussion might also enhance participants’ mindfulness levels in the DG.

Examination of the group differences of the posttest outcome variables suggested that there were medium to large effect sizes of group membership effects on posttest levels of depression and anxiety. These results were consistent with previous studies which confirmed that interventions with mindfulness-based components exerted significant benefits in comparison with control conditions on levels of mindfulness, depression, and anxiety, with small to medium effect sizes (Hofmann et al., 2010; Spijkerman et al., 2016).

Results of pairwise comparisons in the present study suggested that only the GMBI group had significant effects on the outcome variables compared with BCG. Groups of SDMBI and DG did not show significant differences in the outcome variables compared with BCG. The GMBI seemed to exert the strongest effect on the outcome variables compared with other groups. These results suggested that the online MBI in a group approach might enhance the effectiveness of online MBIs. The findings were also in line with previous studies which confirmed that GMBI had greater effect on psychological distress than self-help MBIs (Cavanagh et al., 2013).

Although the SDMBI showed significant pretest and posttest differences on outcome variables, it did not show significant group differences on outcome variables compared with BCG. These results suggested that self-direct MBIs might not be the best choice due to the lack of group support which usually occurs in group-based interventions (Lewis et al., 2012). What’s more, in SDMBI without direct instruction and group discussion, participants were lack of in-depth understanding about mindfulness. In the present study, the online SDMBI included self-help audio guides and reading materials, but these resources seems not enough. The effectiveness of self-guided programs is distinct in terms of varied content, delivery, and guidance (Lewis et al., 2012). More presentation of multimedia such as video guide and smart phone apps might increase the efficiency of SDMBI (Lewis et al., 2012). Another possible reason is that the 8-week time is difficult for participants to remain engagement. One previous study of Cavanagh et al. (2013) conducted a brief self-direct online MBI lasing for 2 weeks, which results supported the feasibility and effectiveness of shorter self-guided MBI. The discrepancy might also be accounted for by our outcomes referring to random control trail reporting the group and pre-post analysis, where in previous studies, one open trail only reports pre-post analysis (Krusche et al., 2012). Also, this study included general population, where previous study of Cavanagh et al. (2013) only included university students who might have better understanding about mindfulness in SDMBI.

The GMBI and SDMBI showed significant pretest-posttest decreases in the total score of emotion regulation difficulties, while the DG did not show significant pre- and post-test effect on emotion regulation difficulties. These results suggested that mindfulness intervention compared with group discussion might serve as a more adaptive approach to improve effective emotion regulation strategies. Group discussion might help cultivate a supporting environment, but experience of social support as non-specific therapeutic factors may not directly improve participants’ adaptive strategies to deal with negative emotions. Mindfulness intervention emphasizes improvement of the present attention to emotions, facilitates self-control ability of emotion impulse, and helps cultivate the acceptance of emotions, all of which help reduce emotion regulation difficulties (Goldin and Gross, 2010; Gratz and Tull, 2010).

Although the result did not show significant group effect on emotion regulation difficulties, the effect size was medium. The small sample in the present study might be a possible reason for the non-significant group effect on emotion regulation difficulties. Our findings also suggested that the relationship between score changes of sub-dimensions of mindfulness (Observing and Describing) and changes in psychological distress across groups could be mediated through the changes in emotion regulation difficulties across groups. These results indicated that possible changes in mindfulness across groups were associated via changes in emotion regulation to improved psychological distress, which were in support with several previous studies showing that dispositional mindfulness was related to psychological distress through the mediating role of emotion regulation difficulties (Pepping et al., 2013, 2016). There may be overlap between conceptions of mindfulness and DER, because these two conceptions both include element of emotional awareness. More research is needed to use other different measures such as emotion regulation scale developed by Gross and John (2003) to confirm our results.

### Limitations and Future Research Directions

Some limitations of the present study and implications for future research should be noted. First, the study was limited by the small sample size, and which was selected from the general population. Therefore, further work needs to be done to determine whether the results could be generalized to clinical samples. Participants in the present study did not display a notable level of psychological symptoms which might have influenced their motivation to participate in the program and to persist through the weekly home practice. This might have in turn reduced the sensitivity of changes in the outcome measures. Future studies could compare the effectiveness of clinical utility of online MBIs with non-clinical populations. Second, the follow-up data was not available in the present study, thus prevented us from investigating the lasting effect of the online MBIs. Future long-term research should examine whether the effects of online MBIs on mindfulness, emotion regulation, depression, and anxiety are maintained over time. Another limitation is that we obtained data of outcome variables by self-report questionnaires. A wider range of assessment resources such as physiological index may be used in future studies. Additionally, future online SDMBI development should explore approaches to optimize the program delivery and maximize acceptability, engagement and effectiveness of online SDMBI. Further research is also needed to identify for whom the SDMBI is likely to be most beneficial as self-interventions are not appropriate for everyone (Lewis et al., 2012), and it’s necessary to explore the individual factors that influence the effectiveness of SDMBI.

## Ethics Statement

This study was carried out in accordance with the recommendations of Code of Ethics for Counseling and Clinical Practice, Chinese Psychological Society. The protocol was approved by the Ethics Committee, Beijing Key Laboratory of Learning and Cognition, Capital Normal University. All subjects gave written informed consent in accordance with the Declaration of Helsinki.

## Author Contributions

YM and ZS contributed equally to this paper. YM mainly contributed to the design of the study and writing of the manuscript. ZS mainly contributed to the design of the study and conducted the intervention. AS, XZ, and XL critically reviewed and revised the manuscript. All authors were accountable for the final version of the manuscript.

## Conflict of Interest Statement

The authors declare that the research was conducted in the absence of any commercial or financial relationships that could be construed as a potential conflict of interest.
